# *Geminiviridae* and *Alphasatellitidae* Diversity Revealed by Metagenomic Analysis of Susceptible and Tolerant Tomato Cultivars across Distinct Brazilian Biomes

**DOI:** 10.3390/v16060899

**Published:** 2024-06-01

**Authors:** Izaías Araújo de Oliveira, Luciane de Nazaré Almeida dos Reis, Maria Esther de Noronha Fonseca, Felipe Fochat Silva Melo, Leonardo Silva Boiteux, Rita de Cássia Pereira-Carvalho

**Affiliations:** 1 Department of Plant Pathology, University of Brasília (UnB), Brasília 70910-900, DF, Brazil; eng.agro16@gmail.com (I.A.d.O.); lucianealmeidareis@outlook.com (L.d.N.A.d.R.); ffochatsm@hotmail.com (F.F.S.M.); 2 Embrapa Vegetable Crops (Hortaliças), National Center for Vegetable Crops Research (CNPH),Brasília 70275-970, DF, Brazil; maria.boiteux@embrapa.br

**Keywords:** breeding, *Solanum lycopersicum*, high-throughput sequencing, single-stranded DNA viruses, tolerance

## Abstract

The diversity of *Geminiviridae* and *Alphasatellitidae* species in tomatoes was assessed via high-throughput sequencing of 154 symptomatic foliar samples collected from 2002 to 2017 across seven Brazilian biomes. The first pool (BP1) comprised 73 samples from the North (13), Northeast (36), and South (24) regions. Sixteen begomoviruses and one *Topilevirus* were detected in BP1. Four begomovirus-like contigs were identified as putative novel species (NS). NS#1 was reported in the semi-arid (Northeast) region and NS#2 and NS#4 in mild subtropical climates (South region), whereas NS#3 was detected in the warm and humid (North) region. The second pool (BP2) comprised 81 samples from Southeast (39) and Central–West (42) regions. Fourteen viruses and subviral agents were detected in BP2, including two topileviruses, a putative novel begomovirus (NS#5), and two alphasatellites occurring in continental highland areas. The five putative novel begomoviruses displayed strict endemic distributions. Conversely, tomato mottle leaf curl virus (a monopartite species) displayed the most widespread distribution occurring across the seven sampled biomes. The overall diversity and frequency of mixed infections were higher in susceptible (16 viruses + alphasatellites) in comparison to tolerant (carrying the *Ty*–1 or *Ty*–3 introgressions) samples, which displayed 9 viruses. This complex panorama reinforces the notion that the tomato-associated *Geminiviridae* diversity is yet underestimated in Neotropical regions.

## 1. Introduction

The *Geminiviridae* is the major family of plant-infecting, insect-transmitted, circular, and single-stranded DNA (ssDNA) viruses [1]. This family comprises ≈ 520 species assigned to 14 highly divergent genera, including *Becurtovirus, Begomovirus*, *Capulavirus*, *Curtovirus*, *Eragrovirus*, *Grablovirus, Mastrevirus*, *Topocuvirus*, *Turncurtovirus*, *Citlodavirus, Maldovirus, Mulcrilevirus, Opunvirus*, and *Topilevirus* [1]. Begomoviruses (genus *Begomovirus*) are transmitted under natural conditions by a complex of cryptic *Bemisia tabaci* species (family Aleyrodidae, order Hemiptera). The *Begomovirus* is the largest genus (with ≈ 445 viruses), encompassing monopartite (with a single DNA–A segment) and bipartite (with DNA–A and DNA–B segments) species [2]. Currently, all newly assigned *Begomovirus* species must have a nucleotide identity of less than 91% to the entire DNA–A segment in relation to isolates of other previously described species within the genus [3].

The DNA–A component of the New World begomoviruses comprises six open reading frames (ORFs): one in the viral sense (AV1) and five in the complementary sense (AC1 to AC5). The AV1 gene codes for the coat protein (CP). The AV2 gene is present only in Old World begomoviruses and codes for the movement protein (MP) [2]. The AC1 gene codes for a protein involved in viral replication (REP), while the AC2 gene is responsible for coding the transcription activating protein (TrAp). The AC3 gene encodes REN, a protein that enhances viral replication [4] and the gene product of the AC4 gene is associated with the expression of symptoms [5]. The AC5 gene codes for a protein related to viral pathogenicity able to suppress the host post-transcriptional gene silencing [6]. Recently, new ORFs were identified in the DNA–A component, including ORF AV3, which codes for a 7.4 KDa protein without ascribed function [7]; ORF AC6, which codes for a protein that plays a role in targeting the host mitochondria [8]; and ORF AC7, which codes for a protein that interacts with AV2 and AC2 proteins, inhibiting RNA silencing and acting as a pathogenicity factor [9]. On the other hand, the DNA–B component comprises the ORF BV1 (=NSP) coding for the nuclear shuttle protein and ORF BC1 (=MP) coding for the movement protein of New World begomoviruses [10].

Bipartite genomes share a common region (CR) of ≈200 nucleotides with conserved motifs (iterons) involved in viral replication [11]. Within the CR is located a conserved nonanucleotide sequence (TAATATTAC) that corresponds to the site of origin of viral replication responsible for Rep binding [11]. Cognate iterons are invariable among DNA–A and DNA–B components of the same virus [12]. Another conserved Rep domain interacts with the plant retinoblastoma protein, being crucial for modulating the host gene expression [13]. Promoter regions (homologous to the ones of the CPs from New World begomoviruses) display nearly palindromic DNA sequences with a conserved core (ACTT–N7–AAGT), which is distinct from that of the Old World begomoviruses [14]. Some begomoviruses also present associations with DNA satellites, which can either attenuate or intensify the symptom expression depending on the relationship between the satellite and its helper virus [15,16,17]. 

The first reports of tomato (*Solanum lycopersicum* L.) diseases induced by begomoviruses in Brazil were carried out in the 1960s and 1970s, including the characterization of tomato golden mosaic virus (TGMV), the first Neotropical species [18]. During this period, begomoviruses occurred only sporadically with no major economic importance. However, this scenario changed after the invasion of the whitefly *B. tabaci* MEAM 1 in the 1990s, resulting in an explosion of regional outbreaks and a substantial emergence of novel begomoviruses [19]. Tomato is a major crop in Brazil, being cultivated across all major biomes, including the warm and humid Amazon Forest; *Caatinga* (semi-arid scrubland); temperate Southern fields; highland and lowland *Cerrado* (Savannah) areas; Atlantic Rain Forest; *Pantanal* (floodplain area); the warm/lowland seashore zone; and the peculiar transition zones of Amazon Forest–*Caatinga*, *Cerrado*–*Caatinga*, and Amazon Forest–*Cerrado* (Supplementary Figure S1). However, little is yet known about the diversity of tomato-infecting *Geminiviridae* and *Alphasatellitidae* across each of these biomes.

In the past decade, metagenomic approaches have facilitated the discovery and identification of many novel and highly divergent members of the *Geminiviridae* family [1]. Metagenomics has also been a fundamental tool to provide a more accurate panorama about the diversity of tomato-infecting ssDNA viruses under Brazilian conditions [20], allowing the detection of ≈ 30 begomoviruses in association with this vegetable crop in the country [20,21].

The employment of resistant cultivars is the most efficient strategy for the management of New World and Old World begomoviruses in tomatoes [22,23,24,25,26]. Two distinct introgression events [27] involving a segment of chromosome 6 of *Solanum chilense* (named as *Ty*–1 and *Ty*–3 genes) allowed the development of cultivars with suitable levels of tolerance [28]. The *Ty*–1 gene (and its putative allele *Ty*–3) encodes for an RDRy-type RNA polymerase [27], being effective against a wide array of begomoviruses [27]. For this reason, the *Ty*–1 and *Ty*–3 introgressions are massively employed in tomato breeding programs worldwide [29]. Interestingly, a putative ‘filtering effect’ of the tolerance factor *Ty*–1 on the diversity of begomoviruses in tomato crops has been observed in Central Brazil in HTS-based surveys [20,21].

In the present work, a broader geographical (across seven Brazilian biomes) and chronological (from 2002 to 2017) survey of the diversity of the tomato-associated *Geminiviridae* species and their satellite DNAs was conducted via an HTS-based approach. The present survey covered samples from the main tomato-producing areas, located in different biomes across all five macro-regions of Brazil. From the breeding standpoint, the present work represents a more extensive sampling of the diversity of these viral pathogens and investigates the potential impact of two tolerance factors (*Ty*–1 and *Ty*–3) on the composition and dynamics of tomato-associated *Geminiviridae* populations.

## 2. Material and Methods

### 2.1. Leaf Samples from Tomato Plants with Begomovirus-Like Symptoms

One hundred and fifty-four (154) leaf samples from tomatoes exhibiting typical begomovirus symptoms (mosaics, leaf deformation, and mottle) were obtained from field surveys in tomato-producing areas in five macro-regions across seven different Brazilian biomes from 2002 to 2017 (Supplementary Table S1). The five macro-regions were North (13), Northeast (36), Central–West (42), Southeast (39), and South (24) (Supplementary Table S1). These samples were selected to cover a broader geographical and chronological snapshot.

### 2.2. DNA Extraction and Molecular Marker Confirmation of the Presence of the Ty–1 and Ty–3 Introgressions

Foliar samples were stored in a freezer (–20 °C) and the total DNA was extracted from them using a modified protocol 2X CTAB plus organic solvents as described [30]. The quantification of the DNA of the samples was carried out using a NanoDrop-1000 spectrophotometer (Thermo Fisher Scientific, Waltham, MA, USA), and the nucleic acid integrity was assessed by electrophoresis (1% agarose gel). Total DNA from leaf samples was used as a template (40 ng/μL) in PCR reactions using specific primers for genomic regions encompassing the codominant molecular markers linked to the *Ty*–1 [31] and *Ty*–3 [27] introgressions. The PCR products were analyzed via electrophoresis (1% agarose gel), stained with ethidium bromide, and visualized under UV light.

### 2.3. Enrichment of Circular DNAs by Rolling Circle Amplification—RCA and Confirmation of Begomovirus Infection

DNA extracted from each individual sample (40 ng/μL) was used as a template for RCA—Rolling Circle Amplification [32]. The confirmation of begomovirus infection in the individual samples (40 ng/μL) was performed essentially as described [33], using the two degenerated primer pairs (PAL1v1978/PAR1c496 and PBL1v2040/PRCc1), targeting conserved regions of the DNA–A component and DNA–B components, respectively [33].

### 2.4. Preparation of Pools of Samples to High-Throughput Sequencing (HTS)

The RCAs of the samples were grouped into two pools (named as BP1 and BP2). Pool BP1 encompassed samples from the North (13), Northeast (36), and South (24) regions, while pool BP2 was composed of samples from the Southeast (39) and Central–West (42) regions (Supplementary Table S1). After establishing the pools, the corresponding libraries were sequenced on an Illumina NovaSeq-6000 (Agrega, Porto Alegre, RS, Brazil) and 150 bp paired-end reads were generated.

### 2.5. Viral Sequence Analyses

The adapter sequences were removed from the HTS data, and the trimmed sequences were subjected to de novo assembly using the CLC Genomics Workbench 23.0.1 program (Qiagen, Hilden, Germany), with the default parameters. The contigs were subsequently analyzed with the Geneious^®^ 11.1 program [34]. All contigs were compared with the viral RefSEq database available at NCBI (https://www.ncbi.nlm.nih.gov; accessed on 3 April 2024), using the BLASTn and BLASTx algorithms. The procedure was carried out essentially as previously described [20,35]. The read files provided by HTS were mapped to virus-like contigs to obtain the final sequence. The information of each individual contig was extended with the help of the Geneious^®^ 11.1 program using the ‘Map to reference’ tool (90 to 99% minimum overlap identity parameter). MUSCLE alignments were performed in Geneious^®^ 11.1 and used for ORF annotation. After de novo assembly, taxonomic prediction analyses were carried out with the contigs from both pools using the Kaiju web server (http://Kaiju.binf.ku.dk/server, accessed on 3 April 2024) [36], with the standard classification parameters. From these analyses, the sequences predicted to be of viral origin were recognized. The largest sequences were selected and assembled. The viral sequences were aligned with the reference genomes showing greater identities [36] with the help of the Geneious^®^ R11.1 program. This program was also used to assemble the viral genome, annotation, and sequence alignments. For potential new viral/subviral species, in addition to the ORF annotation, the intergenic region (present in monopartite begomoviruses) and the common region (present in bipartite begomoviruses) were also analyzed. In the common region, the nonanucleotide motifs and iterons were characterized as well as the Rep Iteron-related domains (REP-IRDs), which allowed us to confirm that pairs of DNA–A and DNA–B components were cognate [14,15]. For comparison across isolates and viral species, the sequences were aligned using pairwise MUSCLE multiple alignment with the help of the Sequence Demarcation Tool (SDT) program [37].

### 2.6. Detection of Viruses in Individual Samples by PCR with Virus-Specific Primers

Based on the sequences obtained by HTS sequencing, open and oppositely directed primer pairs were developed using the primer design function of the Geneious^®^ R11.1 program [34]. The primers were used to detect viruses in individual samples using a row per column system. Sets of virus-specific primers were used to detect viral species in the samples (Supplementary Table S2).

### 2.7. PCR Conditions Used to Detect ssDNA Viruses and Subviral Agents in Individual Samples within Each Pool

PCR assays with species-specific primers were used to recover the viral genomes in each individual DNA sample. Reactions were carried out in a total volume of 12.5 μL, containing the following components: a *Taq* polymerase buffer (10×; 1.25 μL), 50 mM MgCl_2_ (40 μL), 2.5 mM dNTPs (0.25 μL), 10 μM forward and reverse primers (0.25 μL), Milli-Q water (8.0 μL), and *Taq* polymerase 0.5 U (0.10 μL). The 35 amplification cycles were divided into the following steps: initial denaturation (94 °C for 3 min), denaturation (94 °C for 30 s), annealing (see temperatures in Supplementary Table S2) for 45 s, extension (72 °C for 3 min), and final extension (72 °C for 10 min). The amplicons generated were visualized in agarose gel (1%) stained in ethidium bromide and under UV light in a transilluminator and photodocumented.

### 2.8. Validation via Sanger Dideoxy Termination Sequencing of the Amplicons Obtained with Species-Specific PCR Primers

To validate the PCR primers used in virus-specific detection assays (Supplementary Table S2), the amplicons generated by each primer pair were purified using a DNA purification kit (Ludwig Biotech, Alvorada, RS, Brazil) and then subjected to Sanger dideoxy termination sequencing at ACTGene Análises Moleculares (Alvorada, RS, Brazil). The chromatograms were evaluated for their quality and further analyzed using the BLASTn algorithm. The final sequences were compared to the ones available at the NCBI database (https://www.ncbi.nlm.nih.gov, accessed on 3 April 2024) in order to confirm the ssDNA viral and subviral species present in each sample.

## 3. Results

### 3.1. Viral Diversity in the BP1 Pool (Composed of Samples from North, Northeast, and South Brazilian Regions)

The HTS, conducted on the Illumina NovaSeq-6000 platform, provided DNA viral genomic information of the BP1 pool (composed of tomato foliar samples collected in the North, Northeast, and South regions) with the following raw reads: 7,230,366 reads and 38,575 contigs with 137 of them corresponding to genomic segments of viruses as indicated by the BLASTn analysis. HTS-derived genomic information and assembly of contigs from the BP1 pool allowed the recovery of 15 begomovirus-like genomes, 4 of them classified as putative new species—NS (Table 1 and Table 2). Eleven viruses were previously reported as infecting tomatoes, including the monopartite tomato mottle leaf curl virus—ToMoLCV [38,39], the bipartite tomato severe rugose virus—ToSRV [38,40], and Sida micrantha mosaic virus—SimMV [41]. Among the 11 previously characterized *Begomovirus* species, ToMoLCV displayed the highest read coverage (277,339), followed by ToSRV (227,279), tomato golden leaf distortion virus—ToGLDV (170,461), tomato chlorotic mottle Guyane virus—ToCMoGV (131,012), and Chino de tomate Amazonas virus—ChdTAV (55,010). The additional viruses displayed lower read coverage numbers (Table 1). In relation to the number of isolates, the ToMoLCV displayed the highest number (nine isolates), followed by ToGLDV (three) and SimMV (two), and the other viruses were found only in single isolates (Table 1). Sida yellow blotch virus—SiYBV (contig 6958) was the only formerly described begomovirus that was not yet reported in association with tomato crops in this pool.

As mentioned, four DNA–A segments exhibited identity levels lower than the threshold of 91% nucleotide identity (Table 1), which is the current demarcation criterion for a novel *Begomovirus* species [3]. Putative NS#1 (41,497 reads) shared 90.4% identity with tomato interveinal chlorosis virus—ToICV (NC_038469.1), NS#2 (153,967 reads) shared 90.17% identity with ToMoLCV (MT215005.1), NS#3 (79.920 reads) shared 87.10% with tomato bright yellow mosaic virus—ToBYMoV (NC_038468.1), and NS#4 (24.589 reads) shared 82.80% with ToGLDV (HM357456.2). The HTS results from the BP1 pool (Table 2) also allowed the recovery of complete DNA–B segments. Isolates of ToCMGV displayed the highest coverage (129,354 reads) and the highest number of isolates (= four).

A partial genome related to tomato-associated geminivirus 1 (genus *Topilevirus*) was also recovered (Table 1 and Table 2). A topilevirus-like virus (contig 8679) displayed low reads (82) and only partial genomic sequence (2139 nucleotides) recovery (Table 1). The contig associated with the *Topilevirus*-like partial genome shared a nucleotide identity of 90.27% with tomato-associated geminivirus 1 (TAG1; MN527305.1) (Table 1).

### 3.2. Viral Diversity in the BP2 Pool (with Samples from Southeast and Central–West Regions)

The number of reads of the BP2 pool was in the order of 8,533,058, which generated 34,964 contigs, 145 of them corresponding to putative viral genome segments. HTS-derived genomic information and assembly of contigs allowed the recovery of 14 viruses and/or subviral agents (Table 3 and Table 4). Nine of them matched to previously reported tomato-infecting begomoviruses viz.: Euphorbia yellow mosaic virus—EuYMV [20,42], tomato common mosaic virus—ToCMoV [43], SimMV [41], ToSRV [38,40], tomato rugose mosaic virus—ToRMV [20,24,38], tomato yellow vein streak virus—ToYVSV [44,45], ToMoLCV [38,39], tomato golden vein virus—TGVV [20,45], ToICV [46,47], and tomato yellow net virus—ToYNV [21]. ToRMV displayed the highest number of reads (601,302), followed by ToSRV (590,532 reads), SimMV (431,707), ToMoLCV (412,517), and putative NS#5 (369,619).

Regarding the number of isolates, the ToMoLCV displayed the highest number (eleven), followed by TGVV (six), SimMV (five), and ToSRV, TRMV, and ToCMoV (three isolates of each). The remaining viruses varied from one to two representative isolates (Table 3). ToSRV displayed the highest number of reads (300,551 reads) and sequences (four) among the recovered DNA–B segments (Table 4). The genome of a putative new begomovirus (NS#5) was recovered, sharing 89.03% identity with a TGVV isolate (MN928612.1).

Two members of the *Topilevirus* genus were also recovered: tomato-associated geminivirus 2—TAG 2 (contig 934) and ToALCV (contig 45). Contig 934 shared 97.98% identity with TAG 2 (Table 3). Contigs 185 and 45 shared 100% identity with each other and 85.21% identity with ToALCV and 18,143 reads (Table 3). A minimum nucleotide identity of 78% is the demarcation criterion for a new species in the genus *Topilevirus* [48,49]. It was also possible to recover two species of alphasatellites associated with begomoviruses: a putative new *Alphasatellitidae* species and Euphorbia yellow mosaic alphasatellite. Contig 38 corresponds to the satellite DNA of the *Alphasatellitidae* family (with read coverage of 46,535), while the second satellite DNA recovered was Euphorbia yellow mosaic alphasatellite (with 10,481 reads).

### 3.3. Comparative Viral Diversity: BP1 Pool (North, Northeast, and South Regions) Versus BP2 Pool (Southeast and Central–West Regions)

It was possible to notice in the HTS results a greater diversity in the BP1 pool encompassing samples from the North, Northeast, and South regions (16 viruses) in contrast with the BP2 pool encompassing samples from Southeast and Central–West regions (14 viruses). However, the BP2 virome displayed greater quantities of DNA–B segments (Table 4) in addition to subviral agents (Table 3).

### 3.4. PCR Detection with Species-Specific Primers of Geminiviruses and Subviral Pathogens in Individual Samples of the BP1 and BP2 Pools

#### 3.4.1. Northern Region

In the Northern region of Brazil, PCR using species-specific primers allowed the detection of begomoviruses previously reported in other geographical regions, including ToMoLCV, SimMV, ToCMoGV, and ToYSV (Table 5). ToMoLCV was detected in the states of Amazonas (sample AM–012), Roraima (RR–003), and Tocantins (TO–088). Likewise, SimMV was detected in the states of Amazonas (AM–010), Roraima (RR–003 and RR–004), and Tocantins (TO–045 and TO–046). ToCMoGV, a pathogen reported thus far only in French Guiana [50], was found here in the state of Amazonas (sample AM–035). ToYSV infection was observed in the states of Roraima (samples RR–003 and RR–004) and Tocantins (TO–046). Endemic species were also detected, including ToBYMoV and NS#3, which present in a mixed infection in a sample from the state of Tocantins (designated as TO–167).

#### 3.4.2. Northeast Region

ToMoLCV, SimMV, and NS#1 were the begomoviruses detected in the Northeast region (Table 5). Among these, ToMoLCV was the most prevalent, infecting 23 samples. However, our report is the first confirmation of ToMoLCV infection in tomato plants from the state of Ceará (samples CE–001; CE–011; and CE–012). SimMV was present in two samples from the states of Bahia (BA–100) and Pernambuco (PE–011), while novel species #1 was detected in samples from the states of Ceará (CE–001) and Pernambuco (PE–011 and PE–012) (Table 5). Therefore, the predominance of ToMoLCV isolates in this semi-arid region must be highlighted.

#### 3.4.3. South Region

ToSRV, ToMoLCV, SimMV, and two novel begomovirus species were detected in the South region (Table 5 and Table 6). ToSRV was the most prevalent (sixteen samples), followed by ToMoLCV (nine samples) and only one sample with SimMV. ToSRV and ToMoLCV were present in samples from the states of Santa Catarina, Rio Grande do Sul, and Paraná. SimMV was only present in the state of Paraná (PR–143). NS#2 and NS#4 were detected in the state of Paraná. NS#2 was detected in two samples (PR–173 and PR–174) and NS#4 in a single (PR–144) sample (Table 5).

#### 3.4.4. Southeast and Central–West Regions

Seven begomoviruses, two topileviruses, and one alphasatellite were detected via PCR with virus-specific primers in the pool of tomato samples of the Southeast and Central–West regions (Table 5 and Table 6). ToSRV, ToMoLCV, ToCMoV, TGVV, SimMV, and EuYMV were detected in both regions. In the Southeast region, ToSRV was the most prevalent (26 samples), followed by ToCMoV (19 samples), ToMoLCV (15), TGVV (10), SimMV (2), and EuYMV (2). ToCMoV and TGVV have not been reported as infecting tomatoes in the São Paulo state, as well as the presence of isolates from the topilevirus ToALCV (samples SP–172 and SP–173). For the Central–West region, the most prevalent virus was also ToSRV (26 samples) followed by ToMoLCV (21), SimMV (16), ToCMoV (14), TGVV (12), EuYMV (3), and ToYNV (1 sample). NS#5 was detected in eight samples. In this region, an alphasatellite was also detected in the Federal District (DF–024, DF–027, and DF–057), in addition to the topileviruses TAGV in the state of Goiás (GO–495), and ToALCV (SP–172 and SP–173) (Table 5 and Table 6).

### 3.5. Comparative Diversity of Samples with Versus without the Ty–1/Ty–3 Introgressions

**Figure 1:**
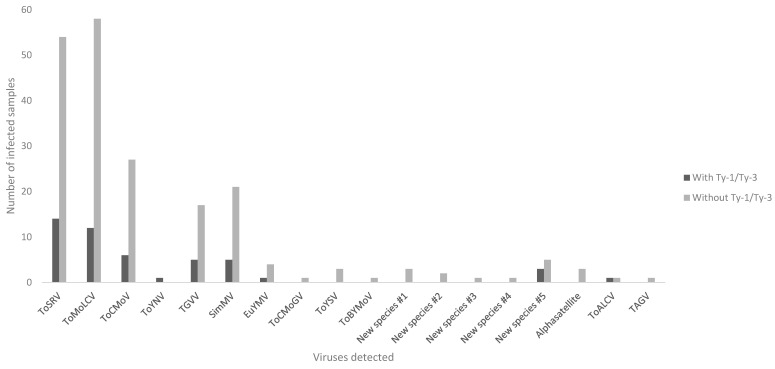
Begomoviruses, Topileviruses, and satellite DNAs detected (X axis) versus the number of tomato samples (Y axis) with the presence and absence of resistance/tolerance factors (*Ty*–1/*Ty*–3). Viruses detected: tomato severe rugose virus (ToSRV), tomato mottle leaf curl virus (ToMoLCV), tomato chlorotic mottle virus (ToCMoV), tomato yellow net virus (ToYNV), tomato golden vein virus (TGVV), Sida micrantha mosaic virus (SimMV), Euphorbia yellow mosaic virus (EuYMV), tomato chlorotic mottle Guyane virus (ToCMoGV), tomato yellow spot virus (ToYSV), tomato bright yellow mottle virus (ToBYMoV), tomato apical leaf curl virus (ToALCV), and tomato-associated geminivirus (TAGV).

**Figure 2:**
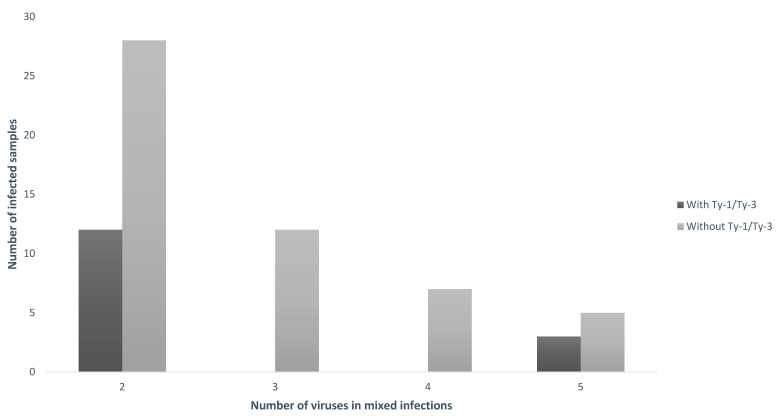
Number of viruses found in mixed infections (X axis) in tomato samples with presence and absence of tolerance factors *Ty*–1/*Ty*–3 (Y axis).

The number of different geminiviruses and associated satellites detected as well as the number of infected samples and the number of mixed infections (Table 5 and Figure 1 and Figure 2) were greater in samples without the *Ty*–1/*Ty*–3 introgressions (Figure 1). Altogether, the number of viruses and subviral agents in susceptible plants was 16 viruses (plus one alphasatellite) versus 9 viruses in plants with the *Ty*–1/*Ty*–3 introgressions (Table 5 and Figure 1).

## 4. Discussion

The most recent worldwide surveys revealed that more than 300 viral species are able to infect the tomato crop [51,52,53,54]. The largest number of tomato-infecting viruses (221) are classified as *Begomovirus* species (family *Geminiviridae*), comprising 66.97% of all viral pathogens reported as infecting this vegetable crop thus far [51,52,53,54]. This scenario of extensive begomovirus diversity is more likely to expand due to genetic plasticity of this group of pathogens, which is generated via mutation, recombination, and pseudorecombination events [55,56].

HTS platforms are allowing the discovery of new ssDNA viruses through virome studies, thus making it possible to monitor the increase in viral diversity across different biomes and over time [20]. We were able to recover genomes of *Begomovirus*, *Topilevirus*, and subviral ssDNA species after a very extensive HTS-based virome of foliar tomato samples was collected across seven Brazilian biomes: the warm and humid Amazon Forest; *Caatinga* (semi-arid scrubland); highland and lowland *Cerrado* (Savannah) areas; Atlantic Rain Forest; the warm/lowland seashore zone; and *Cerrado*–*Caatinga* and Amazon Forest–*Cerrado* transition zones.

The situation of tomato-infecting begomoviruses in Brazil prior to our work indicated a viral complex of more than 26 species [21]. Herein, we potentially added five more tomato-infecting begomoviruses to this pathogenic complex, employing a very representative temporal snapshot of samples (2002 to 2017). These novel begomoviruses will be further characterized via biological and molecular assays. It is important to highlight that our data on the dynamic changes in the relative prevalence across years/geographical areas as well as the discovery of a new set of species gives support to the notion that recurrent surveys must be conducted to provide updated panoramas of tomato-infecting begomoviruses. Our survey also indicated that the diversity of ssDNA viral and subviral species is yet largely underestimated in Neotropical areas. In addition, our results corroborate studies showing the efficiency of HTS for assessment of ‘hidden’ viral richness across different environments and hosts [20,21,57].

The five putative novel begomoviruses detected herein displayed endemic distributions. New *Begomovirus* species #1 was reported in the semi-arid Northeast region, whereas begomoviruses #2 and #4 were collected in mild subtropical climates (South region). NS#3 was detected in the warm and humid (North) region, whereas putative NS#5 was occurring in the continental highland areas (Central–West region). NS#1, detected in the states of Ceará (CE–001) and Pernambuco (PE–011 and PE–012), displayed a DNA–A segment (2604 nts) with 90.40% identity with isolated tomato interveinal chlorosis virus (NC_038469). NS#2 (2631 nts) shared 90.17% identity with ToMoLCV (MT215005) and was detected in the state of Paraná (PR–173 and PR–174). For NS#1 and NS#2, their cognate DNA–B segments were not found, indicating that they are two putative monopartite species. However, more extensive studies searching for these DNA–B cognate segments should be conducted in order to verify their putative monopartite nature. NS#3 (2657 nts) displayed 87.1% with tomato bright mottle virus (NC_038468.1), detected in the state of Tocantins (TO–167). NS#4 displayed 80.2% identity with tomato golden leaf distortion virus (HM357456) and was detected in a single sample in the state of Paraná (PR–144). It has a typical bipartite begomovirus DNA–A segment of 2612 nucleotides (nts), with cognate DNA–B of 2565 nts. Finally, NS#5, detected in the state of Goiás and the Federal District, presented a DNA–A segment of 2561 nts and 89.3% identity with tomato golden vein virus (MN928612.1), its DNA–B segment cognate with 2527 nts. All five new begomovirus species meet the species demarcation criterion of less than 91% identity with other species in the genus [3].

PCR assays with species-specific primer pairs allowed us to verify the presence of novel viruses as well as the geographical dispersion of previously described tomato-infecting begomoviruses across distinct Brazilian regions. Thus far, only four begomoviruses associated with tomato plants were reported in the North region of Brazil [58]. Herein, a new virus was detected in the state of Amazonas, which was previously considered as a begomovirus-free area. We detected ToCMoGV in the AM–035 sample originating from Iranduba (AM) collected in 2016. This virus was already reported in French Guiana [50]. Also, in the North region, ToYSV (=Leonurus mosaic virus) was reported for the first time in tomato plants in the states of Tocantins and Roraima. This ToYSV was detected in the samples TO–046 (collected in 2008 in Gurupi District) and RR–003 and RR–004 (both collected in Boa Vista City in 2013). Until now, reports of ToYSV infecting tomato plants in Brazil were restricted to the Southeast region, in the state of Minas Gerais [59]. The tomato infection by ToMoLCV and SimMV in the North of Brazil is also a novel report. It is worth mentioning that the information on tomato-infecting begomoviruses occurring in the North region (Amazon) is yet very limited, as is the knowledge of viral diversity in this geographic area. Therefore, our study indicates that additional surveys may reveal a peculiar novel set of endemic begomovirus species able to infect tomatoes and other crops.

Thus far, ToMoLCV is the prevalent tomato-infecting begomovirus in the warm and semi-arid Northeast region of Brazil [39,44,46,60], corroborating the results of the present study. However, before our results, there were no reports in the literature of infections in tomato plants by SimMV in the Brazilian Northeast region. SimMV infection, reported here for the first time, can be explained by the great transmission efficiency and the polyphagous habit of the supervector *B. tabaci* [61] as well as by the frequent presence of weeds of the genus *Sida*, which are often in association with commercial tomato cultivation [62,63]. This observation reinforces the epidemiological importance of weeds as a repository and source of inoculum for tomato-infecting viruses [63].

There is an overall lack of information about the panorama of begomovirus on tomatoes in the South region of Brazil, which is composed of three states. Our work is the first report of ToSRV in Rio Grande do Sul and SimMV in Paraná State in association with tomato plants. ToSRV was previously registered in Santa Catarina in the year 2006 [64] and also in Paraná in 2014 [65]. A recent survey in the state of Santa Catarina found that ToSRV is limited to the metropolitan region of Florianópolis [66]. We also provide the first confirmation of tomato plants infected by ToMoLCV across all states of the South regions (Paraná, Rio Grande do Sul, and Santa Catarina). Our study conducted with a relatively low number of samples (24) suggests that the viral diversity associated with tomatoes is likely to be underestimated in this geographic region.

The number of begomoviruses in the Central–West region is very high, corroborating previous studies in this geographic area [20,24,29,43,44,46]. This is the most important geographical region for processing tomato production in the country. We observed a slight prevalence of ToRMV over ToSRV (601,302 versus 590,532 reads) in the Central–West region. However, our results from individual samples confirmed previous surveys that ToSRV is the most prevalent begomovirus in tomato in this area [20,39,40], surpassing ToRMV, whose prevalence was gradually decreasing under natural conditions. ToRMV and ToSRV belong to a complex of bipartite tomato-infecting begomoviruses that share identical iterons. In addition, these viruses have almost identical DNA–B sequences (98.2% identity). Previous studies indicated that ToRMV and ToSRV are able to form pseudorecombinants in tomato plants under experimental conditions in all possible combinations of single and mixed infections [67]. However, there was a preferential detection of both genomic segments from ToRMV over the DNA–A and DNA–B of ToSRV, and the accumulation of ToSRV in mixed infections was reduced compared to that in single infection. In fact, ToSRV shows a high adaptability, infecting a large number of hosts [63] and being present across different regions of the country. These attributes of ToSRV may also explain its prevalence in the Central–West region.

ToSRV, ToMoLCV, ToCMoV, and TGVV were found to be the most prevalent and with wider geographical distribution across the temperate Southeast region. This region is the most important tomato-producing area for the fresh-market consumption in the country and outbreaks of begomoviruses are very often detected across all states [20]. The species ToSRV and ToMoLCV are the most relevant from the tomato breeding standpoint since they were often detected in association with tomato samples with and without the *Ty*–1/*Ty*–3 resistance factors, showing the high adaptive and dissemination capacity of the virus. The lower relative richness of novel begomoviruses outside the Southeast and Central–West regions can be explained by the fact that these regions have been subjected, over the past few decades, to a greater number of prospecting works and surveys of begomovirus diversity via either conventional PCR strategies or via HTS [20,24]. The unequal number of DNA–B segments observed across the pools may allow us to infer the significant use of these segments in pseudorecombination events, allowing viruses to better adapt in the absence of the cognate DNA–B segment and at the same time increase genetic variability.

Although endemic begomoviruses were detected in our survey, no Old World begomoviruses were found in Brazil, suggesting, thus far, the exclusive invasion of non-viruliferous populations of the exotic vector *B. tabaci* MEAM1. The greater number of novel species in the BP1 pool can be explained by a large variation of the landscapes, encompassing distinct ecological niches and biomes. A second hypothesis of the higher number of ssDNA viruses and subviral agents in BP1 could restrict employment of cultivars with either *Ty*–1 or *Ty*–3 introgressions in the sampled regions.

Alphasatellite isolates were detected only in the Federal District, in two adjacent cities of Gama (DF–024 and DF–027) and Ponte Alta (DF–057), revealing that, to date, this agent is endemic to the central region of Brazil. Satellite DNAs are subviral agents that can modulate viral pathogenesis depending on the interaction between the helper virus and the host plant [16,17,68]. The presence of alphasatellites associated with tomato crops was previously reported in the Central–West region of Brazil [20], corroborating the results reported here. A closely related alphasatellite was formally reported in the weeds *Euphorbia heterophylla* (KY559640.1), *Sida* spp. (KX348227.1), and *Cleome affinis*, with either EuYMV or Cleome leaf crumple virus (ClLCrV) as helper viruses [69].

The TAGV (genus *Topilevirus*) was detected in a single sample in Central Brazil (GO–495) collected in 2001 in the city of Planaltina de Goiás (GO). The first report of this topilevirus in tomato plants was also carried out in Central Brazil [70]. However, we detected the presence of the topilevirus ToALCV in Sao Paulo State (Southeast region) in samples SP–172 and SP–173, both originating from Santo Antônio da Posse (SP) in 2015. As far as we know, the presence of ToALCV infecting tomato plants was restricted to the central region of Brazil [71]. The first reports of topileviruses associated with tomato crops were carried out in Brazil [70] and in Argentina [48]. Currently, only two species are reported: tomato-associated geminivirus [70] and tomato apical leaf curl virus [48]. Immediately after the report, tomato apical leaf curl virus (ToALCV) was detected for the first time infecting tomato plants in Central Brazil [71]. Since then, ToALCV has been reported to be associated with tomatoes in other surveys across the Brazilian Central–West area [71]. Analyses based on the amino acids of the CP protein were used to propose that ToALCV can be transmitted by the planthopper *Micrutalis maleifera* (family Membracidae). However, transmission trials have not yet been carried out to confirm this hypothesis [48]. These successive reports of these viruses show the rapid distribution capacity of topileviruses. In fact, the result obtained here represents an expansion in the geographic distribution of the genus since it is the first report outside Central Brazil.

Previous HTS-based surveys revealed that the *Ty*–1 factor might play a role as a “diversity filter”, reducing the number of ssDNA viruses and mixed infections in tomato plants carrying this introgression [20]. Even with an unequal number of samples (121 without and 33 with either *Ty*–1 or *Ty*–3 tolerance factors), the overall diversity observed here was higher in susceptible samples (16 viruses + alphasatellites) in comparison to tolerant samples (9 viruses). In addition, it is interesting to point out that four out of five novel begomoviruses were detected in plants without either *Ty*–1 or *Ty*–3 genes. Overall, these observations are also suggesting a ‘filtering effect’ of both tolerance factors as previously observed [20]. In some cases, species-specific “filtering” was observed for SimMV in the Central–West region, ToSRV in the South, ToMoLCV in the Northeast and Southeast, as well as ToCMoV and TGVV in the Southeast region. However, additional studies should be carried out employing controlled bioassays since local environmental factors (e.g., high temperatures) might interfere with the mRNA and protein expression of these tolerant factors, misleading our conclusion about their full spectrum of efficiency. We also could observe that the number of tomato plants carrying either *Ty*–1 or *Ty*–3 genes displayed lower frequencies of both simple and mixed viral infections. In this regard, our study is the first to assess the impact of the *Ty*–3 gene/allele on the dynamics of the tomato/begomovirus pathosystem.

In addition, our results suggest that viruses that infect tomato plants with these tolerance genes may carry peculiar evolutionary/adaptive processes. For example, NS#5 and a novel isolate of tomato apical leaf curl virus (SP–172) were detected only in tolerant plants exhibiting severe begomovirus-like symptoms. Viruses able to replicate in plants with the *Ty*–1 and *Ty*–3 resistance factors may be undergoing a differential evolutionary/adaptive process, which could result in viral isolates with potentially superior capacity to overcome resistance mediated by these genes. This selective force could be more intense especially for begomoviruses with high adaptability and with greater dispersion and predominance (e.g., ToSRV and ToMoLCV).

## 5. Conclusions

Herein, we uncovered a significant increase in the geographical amplitude of the tomato-begomovirus pathosystem, encompassing different Brazilian biomes as well as geographical regions. Similar to what was previously observed [20], ToSRV (a bipartite species) and ToMoLCV (a monopartite species) were the prevalent begomoviruses in the country, followed by TGVV and ToCMoV. Even though ToMoLCV is predominant in the Northeast region, it is important to highlight that this begomovirus is the most widely distributed, being present across seven biomes across all five macro-geographic regions of Brazil. ToMoLCV is currently reaching areas where ToSRV was not yet able to establish. In addition, when comparing ToSRV and ToMoLCV regarding their ability to infect tomato plants with the presence of *Ty*–1/*Ty*–3 factors, both viruses displayed very similar frequencies in these samples (14 versus 12 detections, respectively).

The adaptation to the *Ty*–1/*Ty*–3 tolerance factors may also be related to the diversity of viruses with RNA genomes that are simultaneously infecting tomato plants with these introgressions. It has already been found that the presence of the tomato chlorosis crinivirus (ToCV) may reduce the efficiency of the *Ty*–1-mediated tolerance to tomato yellow leaf curl virus—ToYLCV in Europe [72]. In this scenario, the use of gene pyramiding of multiple resistance factors against criniviruses [73] and begomoviruses [74] would be a promising strategy for generating phenotypic stable sources of resistance.

In conclusion, we demonstrated the efficiency of HTS-based platforms in combination with virus-specific PCR assays as tools for the large-scale study of the diversity of *Geminiviridae* species across different regions over time. Novel species and novel tomato-begomovirus interactions were detected. The present study also provided new insights on the begomovirus distribution across Brazil and the confirmation of the ToSRV and ToMoLCV as the most prevalent and the most disseminated pathogens as well as with the best adaptation to the *Ty*–1/*Ty*–3 factors. The diversity detected in the susceptible samples (16 viruses + alphasatellites) and the frequency of mixed infections was higher than the ones with tolerance (9 viruses), suggesting that the *Ty*–1/*Ty*–3 genes may interfere with the overall diversity.

This complex panorama reinforces the notion that the *Geminiviridae* diversity is yet underestimated under Neotropical conditions. All these data will help to guide breeding programs regarding the most effective control strategies and to update the status on emergent and consolidated tomato-infecting begomoviruses in Brazil. Hence, we provide a more accurate overview of the current situation of begomoviruses in tomato plants in Brazil that could help the understanding of the population dynamics of these viruses and their behavior in relation to the main tolerance genes used to control these viruses in the country (*Ty*–1/*Ty*–3).

## Figures and Tables

**Table 1:** Code of the contigs, read coverage, assembled genome size, BLASTn coverage, sequence identity of the assembled virus, E-value, virus description, and GeneBank accession number for the DNA–A segment of *Geminiviridae* viruses and subviral agents obtained by High-Throughput Sequencing (HTS) within pool BP1 (containing 73 foliar tomato samples from the North, Northeast, and South regions of Brazil). Contigs highlighted in gray and bold letters represent putative new viral species.

Code of the Contigs	Read Coverage	Assembled Genome Size (nts)	BLASTn Coverage (%)	Identity (%)	E-Value	Virus Description *	GeneBank Accession Number
40	227,279	2593	100	99.96	0	Tomato severe rugose virus DNA–A ^1^	MW573989.1
*	17,827	2660	100	99.85	0	Tomato yellow leaf deformation dwarf virus DNA–A^1^	NC_055586.1
38	131,012	2630	100	99.73	0	Tomato chlorotic mottle Guyane virus DNA–A ^1^	MK878452.1
5	31,367	2694	99	99.52	0	Tomato rugose yellow leaf curl virus DNA–A ^1^	KU682839.1
1098	9959	2661	100	98.99	0	Tomato yellow spot virus DNA–A ^3^	KX348172.1
63	277,339	2634	100	98.97	0	Tomato mottle leaf curl virus DNA–A ^4^	KX896408.1
66	55,010	2603	100	98.47	0	Chino del tomate Amazonas virus DNA–A ^1^	NC_038443.1
7	170,461	2623	99	98.25	0	Tomato golden leaf distortion virus DNA–A ^3^	HM357456.2
125	205,526	2631	100	97.00	0	Tomato mottle leaf curl virus DNA–A ^6^	MT215005.1
144	156,309	2623	99	96.85	0	Tomato golden leaf distortion virus DNA–A ^2^	HM357456.2
140	77,529	2627	100	96.66	0	Tomato mottle leaf curl virus DNA–A ^5^	JF803247.1
44	194,632	2632	100	95.79	0	Tomato mottle leaf curl virus DNA–A ^4^	KX896414.1
552	4407	2686	100	95.38	0	Sida micrantha mosaic virus DNA–A ^2^	KC706535.1
105	152,241	2623	99	95.18	0	Tomato golden leaf distortion virus DNA–A ^1^	HM357456.2
109	14,715	2661	100	95.11	0	Tomato yellow spot virus DNA–A ^1^	KX348172.1
21	44,698	2622	99	94.82	0	Tomato bright yellow mottle virus DNA–A ^1^	NC_038468.1
6958	2854	2643	99	94.54	0	Sida yellow blotch virus DNA–A ^1^	MT103998.1
67	254,814	2631	100	92.49	0	Tomato mottle leaf curl virus DNA–A ^1^	KX896414.1
68	129,337	2631	100	91.66	0	Tomato mottle leaf curl virus DNA–A ^1^	JF803250.1
205	32,299	2685	100	91.01	0	Sida micrantha mosaic virus DNA–A ^1^	EU908733.1
**New species #1**	41,497	2604	100	90.40	0	Tomato interveinal chlorosis virus DNA–A ^2^	NC_038469.1
8679	82	2139	100	90.27	0	Tomato-associated geminivirus 1 ^1^	MN527305.1
**New species #2**	153,967	2631	100	90.17	0	Tomato mottle leaf curl virus DNA–A^1^	MT215005.1
**New species #3**	7992	2657	99	87.10	0	Tomato bright yellow mottle virus DNA–A^1^	NC_038468.1
**New species #4**	24,589	2612	97	82.80	0	Tomato golden leaf distortion virus DNA–A^1^	HM357456.2

* Virus obtained from Kaiju online tool. Viruses with the same superscript number correspond to distinct isolates of the same species.

**Table 2:** Code of the contigs, read coverage, assembled genome size, assembled genome size, BLASTn coverage, sequence identity of the assembled virus, E-value, virus description, and GeneBank accession number for the DNA–B segment of begomoviruses obtained by High-Throughput Sequencing (HTS) within pool BP1 containing 73 foliar tomato samples from the North, Northeast, and South regions of Brazil.

Code of the Contigs	Read Coverage	Assembled Genome Size (nts)	BLASTn Coverage (%)	Identity (%)	E-Value	Virus Description *	GeneBank Accession Number
10	129,354	2593	100	99.92	0	Tomato chlorotic mottle Guyane virus DNA–B ^2^	MK878451.1
98	3725	2609	100	99.00	0	Tomato yellow leaf deformation dwarf virus DNA–B ^2^	NC_060089.1
81	18,916	2535	100	97.09	0	Chino del tomate Amazonas virus DNA–B ^2^	MG675220.1
2034	92,661	2662	83	96.76	0	Tomato chlorotic mottle Guyane virus DNA–B ^1^	MK878451.1
1156	15,163	2634	100	93.70	0	Tomato yellow spot virus DNA–B ^3^	KX348205.1
2913	82,057	2597	100	91.62	0	Tomato chlorotic mottle Guyane virus DNA–B ^1^	MK878451.1
1778	1475	2583	100	88.31	0	Tomato crinkle leaf yellows virus DNA–B ^3^	JN419011.1
299	2808	2619	99	83.98	0	Tomato yellow leaf deformation dwarf virus DNA–B ^3^	NC_060089.1
93	10,358	2565	85	82.05	0	Tomato interveinal chlorosis virus-2 DNA–B ^2^	MK087039.1
8	14,354	2656	96	77.95	0	Tomato rugose yellow leaf curl virus DNA–B ^1^	JN381822.1

* Virus obtained from Kaiju online tool. Viruses with the same superscript number correspond to distinct isolates of the same species.

**Table 3:** Code of the contigs, read coverage, assembled genome size, BLASTn coverage, sequence identity of the assembled virus, E-value, virus description, and GeneBank accession number for the DNA–A segment of *Geminiviridae* viruses and subviral agents obtained by High-Throughput Sequencing (HTS) of pool BP2 containing 81 foliar tomato samples from the Southeast and Central–West regions. Contig highlighted in gray and bold letters corresponds to a putative new viral species.

Code of the Contigs	Read Coverage	Assembled Genome Size (nts)	BLASTn Coverage (%)	Identity (%)	E-Value	Virus Description *	GeneBank Accession Number
38	46,535	1322	100	99.92	0	*Alphasatellitidae* sp.^1^	MT214093.1
107	574,158	2591	100	99.88	0	Tomato severe rugose virus DNA–A ^5^	MT733811.1
78	73,475	2631	100	99.81	0	Tomato mottle leaf curl virus DNA–A ^3^	MT733813.1
255	1205	2628	100	99.51	0	Euphorbia yellow mosaic virus DNA–A ^1^	MN782438.1
52	206,835	2622	100	99.50	0	Tomato chlorotic mottle virus DNA–A ^2^	MT733804.1
79	57,409	2561	100	99.45	0	Tomato golden vein virus DNA–A ^4^	KC706652.1
827	601,302	2698	99	99.32	0	Tomato rugose mosaic virus DNA–A ^1^	MT215006.1
34	113,869	2561	100	98.83	0	Tomato golden vein virus DNA–A ^1^	KC706646.1
14432	113	2636	100	98.79	0	Tomato yellow net virus DNA–A ^2^	MT214096.1
47	12,561	2610	100	98.74	0	Euphorbia yellow mosaic virus DNA–A ^2^	KY559437.1
211	245,699	2631	100	98.29	0	Tomato mottle leaf curl virus DNA–A ^3^	MT733813.1
29	335,413	2606	100	98.05	0	Tomato rugose mosaic virus DNA–A ^2^	MT215006.1
934	1694	2574	100	97.98	0	Tomato-associated geminivirus 2 ^1^	MN527305.1
37	154,963	2631	100	97.95	0	Tomato mottle leaf curl virus DNA–A ^2^	MT214088.1
*	26,055	2560	100	97.89	0	Tomato yellow vein streak virus DNA–A ^1^	KC136337.1
7	232,163	2622	100	97.83	0	Tomato chlorotic mottle virus DNA–A ^3^	MT733804.1
268	4530	2671	99	97.78	0	Sida micrantha mosaic virus DNA–A ^4^	JX415194.1
73	138,272	2561	100	97.77	0	Tomato golden vein virus DNA–A ^4^	JF803259.1
*	4822	2667	100	97.76	0	Sida yellow mosaic virus DNA–A ^1^	AY090558.1
101	264,219	2631	100	96.92	0	Tomato mottle leaf curl virus DNA–A ^5^	MT215005.1
50	286,731	2637	95	96.83	0	Tomato rugose mosaic virus DNA–A ^1^	MT215006.1
74	3857	2560	100	96.76	0	Tomato yellow vein streak virus DNA–A ^1^	MN508216.1
40	229,047	2631	100	95.79	0	Tomato mottle leaf curl virus DNA–A ^4^	MT733813.1
307	94,086	2676	100	95.70	0	Sida micrantha mosaic virus DNA–A ^1^	KC706535.1
201	10,481	1365	100	94.59	0	Euphorbia yellow mosaic alphasatellite ^1^	FN436008.1
49	203,042	2602	100	93.45	0	Tomato chlorotic mottle virus DNA–A ^1^	MT733804.1
23	145,276	2631	100	94.19	0	Tomato mottle leaf curl virus DNA–A ^1^	JF803247.1
*	431,707	2676	100	94.18	0	Sida micrantha mosaic virus DNA–A ^1^	MT214092.1
*	204,035	2618	100	93.81	0	Tomato interveinal chlorosis virus DNA–A ^1^	JF803253.1
56	412,517	2631	100	93.06	0	Tomato mottle leaf curl virus DNA–A ^1^	MT733813.1
3065	123,209	2572	100	92.76	0	Tomato severe rugose virus DNA–A ^1^	HQ606468.1
357	36,971	2675	100	91.76	0	Sida micrantha mosaic virus DNA–A ^1^	MT214092.1
84	21,005	2556	100	91.68	0	Tomato golden vein virus DNA–A ^1^	KC706653.1
**New species #5**	369,619	2561	100	89.03	0	Tomato golden vein virus DNA–A ^1^	MN928612.1
45	18,143	2879	100	85.21	0	Tomato apical leaf curl virus ^2^	MT135209.1

* Virus obtained from Kaiju online tool. Viruses and subviral agents with the same superscript number correspond to distinct isolates of the same species.

**Table 4:** Code of the contigs, read coverage, assembled genome size, BLASTn coverage, sequence identity of the assembled virus, E-value, virus description, and GeneBank accession number for the DNA–B segments of begomoviruses obtained by High-Throughput Sequencing (HTS) of pool BP2 containing 81 foliar tomato samples from the Southeast and Central–West regions of Brazil.

Code of the Contigs	Read Coverage	Assembled Genome Size (nts)	BLASTn Coverage (%)	Identity (%)	E-Value	Virus Description *	GeneBank Accession Number
103	143,673	2571	100	99.73	0	Tomato severe rugose virus DNA–B ^4^	MT215002.1
82	102,342	2597	100	99.58	0	Tomato chlorotic mottle virus DNA–B ^6^	MT214087.1
16	137	2570	100	99.57	0	Tomato rugose mosaic virus DNA–B ^7^	MT215007.1
14	47,649	2533	100	99.33	0	Tomato golden vein virus DNA–B ^3^	MN928611.1
122	48,514	2551	100	98.55	0	Tomato golden vein virus DNA–B ^9^	MT733807.1
132	142,262	2554	100	98.26	0	Tomato severe rugose virus DNA–B ^2^	MT214085.1
121	147,477	2571	93	97.00	0	Tomato severe rugose virus DNA–B ^1^	MT215002.1
*	45,881	2543	100	96.58	0	Tomato mild leaf curl virus DNA–B ^1^	DQ336352.1
27613	4	318	100	96.54	0	Sida micrantha mosaic virus DNA–B ^1^	AJ557452.1
3050	90,728	2597	85	90.55	0	Tomato rugose mosaic virus DNA–B ^1^	MT214091.1
21	37,396	2527	97	95.50	0	Tomato golden vein virus DNA–B ^1^	KC706660.1
499	2740	2556	100	94.97	0	Tomato yellow vein streak virus DNA–B ^1^	MN508217.1

* Virus obtained from Kaiju online tool. Viruses with the same superscript number correspond to distinct isolates of the same species.

**Table 5:** Positive samples for viruses detected via PCR with species-specific primers in tomato cultivars without carrying either *Ty*–1 or *Ty*–3 or both introgression events in samples collected across the five Brazilian regions.

Virus Acronyms * (Number of Positive Samples)	Codes of the Isolates with Positive PCR Detection per Pathogen per Geographical Region				
	North	Northeast	South	Southeast	Central–West
ToSRV (15 + 20 + 19 = 54)	–	–	PR–143; PR–173; PR–174; RS–033; RS–012; RS–013; RS–014; RS–015; SC–001; SC–002; SC–015; SC–030; SC–032; SC–044; and SC–051	MG–013; MG–014; MG–108; MG–109; MG–267; MG–292; SP–003; SP–006; SP–008; SP–017; SP–111; SP–205; SP–206; SP–154; SP–173; SP–201; SP–239; SP–254; SP–259; and SP–274	DF–663; DF–034; DF–209; DF–487; GO–121; GO–033; GO–034; GO–126; GO–127; GO–204; GO–208; GO–211; GO–212; GO–218; GO–589; GO–604; GO–605; GO–617; and GO–618
ToMoLCV (2 + 23 + 9 + 14 + 10 = 58)	AM–012 and RR–003	BA–034; BA–035; BA–050; BA–100; BA–128; BA–134; BA–143; BA–173; BA–174; CE–001; CE–011; CE–012; PB–025; PB–027; PE–027; PE–028; PE–011; PE–012; PE–099; PE–100; PE–104; PE–105; and PE–121	PR–144; PR–173; PR–174; RS–015; RS–071; RS–095; SC–002; SC–015; and SC–030	MG–013; MG–014; MG–109; MG–292; MG–381; SP–003; SP–056; SP–058; SP–111; SP–213; SP–205; SP–154; SP–173; and SP–201	DF–027; DF–024; DF–054; DF–154; DF–487; GO–495; GO–604; GO–605; GO–617; and GO–618
ToCMoV (16 + 11 = 27)	–	–	–	MG–046; MG– 013; MG–014; MG–084; MG–109; MG–267; MG–292; MG–381; SP–004; SP–006; SP–008; SP–017; SP–056; SP–205; SP–239; and SP–254	DF–027; DF–024; DF–034; DF–044; DF–054; DF–057; DF–170; DF–209; GO–121; GO–126; and GO–127
TGVV (8 + 8 = 16)	–	–	–	MG–046; MG–013; MG–014; MG–108; MG–109; SP–003; SP–017; and SP–206	DF–027; DF–024; DF–170; DF–209; GO–121; GO–126; GO–127; and GO–218
SimMV (5 + 2 + 1 + 2 + 11 = 21)	AM–010; RR–003; RR–004; TO–045; and TO–046	BA–100 and PE–011	PR–143	MG–267 and SP–173	DF–024; DF–034; DF–044; DF–054; DF–170; DF–209; GO–121; GO–033; GO–126; GO–127; and GO–204
EuYMV (1 + 3 = 4)	–	–	–	SP–003	DF–170; GO–204; and GO–208
ToCMoGV (1)	AM–035	–	–	–	–
ToYSV (3)	RR–003; RR–004; and TO–046	–	–	–	–
ToBYMoV (1)	TO–167	–	–	–	–
New species #1 (3)	–	CE–001; PE–011; and PE–012	–	–	–
New species #2 (2)	–	–	PR–173 and PR–174	–	–
New species #3 (1)	TO–167	–	–	–	–
New species #4 (1)	–	–	PR–144	–	–
New species #5 (5)	–	–	–	–	DF–209, GO–121, GO–126, GO–127, and GO–218
Alfasatellite (3)	–	–	–	–	DF–024, DF–027, and DF–057
ToALCV (1)	–	–	–	SP–173	–
TAGV (1)	–	–	–	–	GO–495

* Viruses: Tomato severe rugose virus (ToSRV), tomato mottle leaf curl virus (ToMoLCV), tomato chlorotic mosaic virus (ToCMoV), tomato golden vein virus (TGVV), Sida micrantha mosaic virus (SimMV), Euphorbia yellow mosaic virus (EuYMV), tomato yellow spot virus (ToYSV), tomato bright yellow mottle virus (ToBYMoV), tomato apical leaf curl virus (ToALCV), and tomato-associated geminivirus (TAGV).

**Table 6:** Positive samples for geminiviruses detected via PCR with species-specific primers exclusively in tomato cultivars carrying either *Ty*–1 or *Ty*–3 or both introgression events in samples collected across the five Brazilian regions.

Virus Acronyms (Total of Positive Samples)	Codes of the Isolates with Positive PCR Detection per Pathogen per Geographical Region				
	North	Northeast	South	Southeast	Central–West
ToSRV (1 + 6 + 7 = 14)	–	–	PR–112	MG–268; MG–291; SP–018; SP–156; SP–240; and SP–252	DF–216; DF–235; DF–338; DF–530; DF–546; DF–528; and DF–541
ToMoLCV (1+ 11 = 12)	–	–	–	SP–172	DF–155; DF–235; DF–530; DF–546; DF–528; DF–541; GO–124; GO–229; GO–342; GO–499; and GO–526
ToCMoV (3 + 3 = 6)	–	–	–	MG–268; SP–066; and SP–252	DF–216; DF–235; and GO–124
TGVV (1 + 4 = 5)	–	–	–	SP–018	DF–216; DF–235; GO–124; and GO–229
ToYNV (1)	–	–	–	–	GO–342
SimMV (5)	–	–	–	–	DF–216; DF–235; DF–338; GO–005; and GO–124
EuYMV (1)	–	–	–	SP–066	–
New species #5 (3)	–	–	–	–	DF–216, DF–235; and GO–124
ToALCV ToALCV (1)	–	–	–	SP–172	–

Tomato severe rugose virus (ToSRV), tomato mottle leaf curl virus (ToMoLCV), tomato chlorotic mosaic virus (ToCMoV), tomato golden vein virus (TGVV), tomato yellow net virus (ToYNV), Sida micrantha mosaic virus (SimMV), Euphorbia yellow mosaic virus (EuYMV), tomato yellow spot virus (ToYSV), and tomato apical leaf curl virus (ToALCV).

## Data Availability

No new data were created.

## Supplementary Materials

The following supporting information can be downloaded at: https://www.mdpi.com/article/10.3390/v16060899/s1, Figure S1: Map illustrating the major Brazilian biomes.; Table S1: Information on geographical regions, absence and/or presence of the molecular markers associated with the resistance factors *Ty*–1 and *Ty*–3, year of collection and code of the 154 leaf samples of tomato cultivars (*Solanum lycopersicum*) used in the present study.; Table S2: List of species-specific primers used to detect different viruses and satellite DNA in tomato samples and details of information regarding the name of the primer, sequences, and annealing temperatures (AT ºC).
