# Supplementary material for: Geminiviridae and Alphasatellitidae Diversity Revealed by Metagenomic Analysis of Susceptible and Tolerant Tomato Cultivars across Distinct Brazilian Biomes
Source: Viruses. 2024 Jun 1;16(6):899. doi: 10.3390/v16060899 (PMC11209153; doi:10.3390/v16060899)
Supplement: Supplementary file 1 [file viruses-16-00899-s001.zip › Supplementary table S1.pdf]

Supplementary Table S1. Information on geographical regions, absence and/or presence of the molecular markers associated with the resistance factors *Ty*–1 and *Ty*–3, year of collection and code of the 154 leaf samples of tomato cultivars (*Solanum lycopersicum*) used in the present study. Samples were collected across all five Brazilian regions and all of them displayed variable levels of begomovirus-like symptoms.

Pool	Regions/Biomes	*Ty*-1	*Ty*-3	Year of colletion	Isolate code	City	Geographic coordinates
**BP1**	North/Amazon + Amazon-*Cerrado* transition	Absent	Absent	2005	TO–026	Colméia	3°05'00"S 59°59'44"W
				2007	AM–010	Silves	2°51'04"S 58°34'35"W
					AM–012	Silves	2°51'04"S 58°34'35"W
				2008	TO–083	Araguaina	7°12'39"S 48°13'02"W
					TO–167	Araguaina	7°12'39"S 48°13'02"W
					TO–045	Gurupi	11°44'06"S 49°05'08"W
					TO–046	Gurupi	11°44'06"S 49°05'08"W
					TO–094	Aragominas	7°10'00"S 48°31'32"W
					TO–095	Aragominas	7°10'00"S 48°31'32"W
				2013	RR–003	Boa Vista	2°48'25"N 60°41'47"W
					RR–004	Boa Vista	2°48'25"N 60°41'47"W
				2016	AM–035	Iranduba	3°16'26"S 60°11'02"W
					AM–037	Iranduba	3°16'26"S 60°11'02"W
	North-East/ Semi-arid region + Semi-arid-*Cerrado* transition	Present		2010	BA–063	Jaguaquara	13°31'54"S 39°58'08"W
					CE–046	Guaraciaba do Norte	4°09'52"S 40°45'09"W
					CE–048	Croatá	4°22'40"S 40°58'12"W
					CE–049	Croatá	4°22'40"S 40°58'12"W

				2011	BA–124	Utinga	12°04'49"S 41°05'50"W
				2014	PE–122	Floresta	17°06'12"S 44°29'30"W
				2016	PE–123	Senharó	8°22'16"S 36°32'15"W
		Absent		2005	CE–001	Guaraciaba do Norte	4°09'52"S 40°45'09"W
					CE–011	Carnaubal	4°09'53"S 40°56'24"W
					CE–012	Carnaubal	4°09'53"S 40°56'24"W
					PE–027	Petrolina	9°25'14"S 40°25'27"W
					PE–028	Petrolina	9°25'14"S 40°25'27"W
				2007	BA–034	América Dourada	11°27'13"S 41°26'13"W
					BA–035	América Dourada	11°27'13"S 41°26'13"W
				2009	BA–050	Igarachi	13°20'05"S 42°01'17"W
					PE–011	Pesqueira	8°25'47"S 36°45'11"W
					PE–012	Pesqueira	8°25'47"S 36°45'11"W
				2011	BA–100	Jaguaquara	13°31'54"S 39°58'08"W
					BA–128	Wagner	12°16'57"S 41°10'21"W
					BA–134	Wagner	12°16'57"S 41°10'21"W
					BA–143	Irecê	11°18'25"S 41°51'00"W
					CE–052	Guaraciaba do Norte	4°09'52"S 40°45'09"W
					CE–053	Guaraciaba do Norte	4°09'52"S 40°45'09"W
					CE–057	São Benedito	4°02'52"S 40°52'05"W
					CE–058	São Benedito	4°02'52"S 40°52'05"W

				2012	PE–099	Pesqueira	8°25'47"S 36°45'11"W
					PE–100	Pesqueira	8°25'47"S 36°45'11"W
					PE–104	Poção	8°13'07"S 36°42'43"W
					PE–105	Poção	8°13'07"S 36°42'43"W
				2014	BA–173	Poções	14°34'13"S 40°25'18"W
					BA–174	Poções	14°34'13"S 40°25'18"W
					PE–121	Floresta	17°06'12"S 44°29'30"W
				2016	CE–072	Guaraciaba do Norte	4°09'52"S 40°45'09"W
					CE–073	Guaraciaba do Norte	4°09'52"S 40°45'09"W
					PB–025	Camalaú	7°53'17"S 36°49'27"W
					PB–027	Camalaú	7°53'17"S 36°49'27"W
	South/Atlantic Rain Forest + warm lowland seashore zone		Present	2006	PR–112	Faxinal	23°59'46"S 51°19'18"W
			Absent	2005	RS–033	Feliz	29°27'18"S 51°18'19"W
					PR–111	Faxinal	23°59'46"S 51°19'18"W
					SC–001	Florianópolis	27°34'17"S 48°28'13"W
					SC–002	Florianópolis	27°34'17"S 48°28'13"W
					SC–015	Santo Amaro da Imperatriz	27°41'20"S 48°45'26"W
				2008	PR–079	Reserva	24°39'13"S 50°51'09"W
					SC–030	Caçador	26°06'45"S 49°30'56"W
					SC–032	Caçador	26°06'45"S 49°30'56"W
				2009	RS–012	Dom Pedro de Alcântara	29°22'18"S 49°51'10"W

					RS–013	Dom Pedro de Alcântara	29°22'18"S 49°51'10"W
					RS–014	Torres	29°20'31"S 49°43'56"W
					RS–015	Torres	29°20'31"S 49°43'56"W
				2010	SC–034	Caçador	26°06'45"S 49°30'56"W
				2011	RS–040	Caxias do Sul	29°09'55"S 51°10'24"W
					RS–045	Lajeado Grande	29°04'29"S 50°36'21"W
					SC–044	Santo Amaro da Imperatriz	27°44'43"S 48°47'40"W
					SC–051	Santo Amaro da Imperatriz	27°44'43"S 48°47'40"W
				2013	RS–071	Morro Redondo	31°35'05"S 52°38'31"W
				2015	PR–143	Mauá da Serra	23°53'56"S 51°13'31"W
				2015	PR–144	Mauá da Serra	23°53'56"S 51°13'31"W
				2016	RS–095	Nova Pádua	29°01'40"S 51°18'37"W
				2017	PR–173	Bandeirantes	29°01'40"S 51°18'37"W
					PR–174	Bandeirantes	29°01'40"S 51°18'37"W
**BP2**	South-East / Atlantic Rain Forest + warm lowland seashore zone + *Cerrado* (Savannah) area	Present		2001	SP–066	Sumaré	22°50'30"S 47°15'39"W
				2007	SP–018	Mogi guaçu	22°14'01"S 47°00'59"W
				2010	MG–268	Mateus Leme	20°00'10"S 44°25'48"W
				2011	MG–291	Ituiutaba	18°59'07"S 49°31'36"W
				2014	SP–156	Itapeva	23°58'55"S 48°52'55"W
				2015	SP–172	Santo Antonio da Posse	22°36'16"S 46°54'55"W
				2016	SP–240	Mogi Guaçu	22°14'01"S 47°00'59"W

					SP–252	Mogi Guaçu	22°14'01"S 47°00'59"W
					SP–265	Sebastianópolis do Sul	20°39'29"S 49°55'27"W
			Present		SP–260	Itarana	19°52'21"S 40°52'30"W
		Absent	Absent	2001	MG–046	Ituiutaba	18°59'07"S 49°31'36"W
				2002	MG–013	Serra Negra	20°02'27"S 43°56'35"W
					MG–014	Serra Negra	20°02'27"S 43°56'35"W
				2003	SP–003	Monte Mor	22°55'59"S 47°17'07"W
				2003	SP–004	Monte Mor	22°55'59"S 47°17'07"W
				2006	SP–006	Jaguariúna	22°41'26"S 46°59'15"W
					SP-008	Jaguariúna	22°41'26"S 46°59'15"W
				2007	SP–017	Mogi guaçu	22°14'01"S 47°00'59"W
				2008	SP–056	Piracicaba	22°47'29"S 47°27'59"W
					SP–058	Piracicaba	22°47'29"S 47°27'59"W
				2010	MG–084	Capão Bonito	24°01'12"S 48°17'57"W
					MG–108	Capão Bonito	24°01'12"S 48°17'57"W
					MG–109	Capão Bonito	24°01'12"S 48°17'57"W
					MG–267	Mateus Leme	20°00'10"S 44°25'48"W
				2011	MG–292	Ituiutaba	18°59'07"S 49°31'36"W
					SP–111	Capão Bonito	24°01'12"S 48°17'57"W
					SP–124	Sumaré	22°50'30"S 47°15'39"W
				2013	SP–213	Bragança Paulista	22°55'44"S 46°33'45"W

				2014	SP–205	Araçoiaba da Serra	23°37'53"S 47°29'48"W
					SP–206	Araçoiaba da Serra	23°37'53"S 47°29'48"W
					SP–154	Itapeva	23°58'55"S 48°52'55"W
				2015	MG–381	Itacarambi	15°05'44"S 44°05'52"W
					SP–173	Santo Antonio da Posse	22°36'16"S 46°54'55"W
					SP–201	Serra Negra	20°02'27"S 43°56'35"W
				2016	SP–230	Serra Negra	20°02'27"S 43°56'35"W
					SP–239	Mogi Guaçu	22°14'01"S 47°00'59"W
					SP–254	Mogi Guaçu	22°14'01"S 47°00'59"W
					SP–259	Itarana	19°52'21"S 40°52'30"W
					SP–274	Guapiara	24°12'45"S 48°32'44"W
	Central-West / High land and lowland *Cerrado* (Savannah) area	Present		2002	GO–005	Inhumas	16°22'24"S 49°29'37"W
				2003	GO–124	Goianápolis	16°30'24"S 49°01'02"W
		Absent	Present		GO–229	Morrinhos	17°43'50"S 49°07'02"W
			Present	2005	DF–155	Vargem Bonita	15°55'02"S 48°03'02"W
			Present		DF–216	Taquara	15°37'51"S 47°31'09"W
			Present	2006	GO–342	Itaberai	16°01'41"S 49°47'47"W
			Present	2007	DF–235	Planaltina	15°52'33"S 47°12'00"W
		Present	Present	2010	DF–338	Planaltina	15°52'33"S 47°12'00"W
			Present	2011	GO–499	Planaltina	15°27'21"S 47°36'11"W
			Absent	2012	GO–526	Corumbá de Goiás	15°55'28"S 48°48'24"W

				2013	DF–530	Sobradinho	15°37'52"S 47°51'33"W
					DF–546	Pipiripau	15°36'08"S 47°39'56"W
					DF–541	Paranoá	15°53'19"S 47°33'36"W
		Absent	Present		DF–528	Sobradinho	15°37'52"S 47°51'33"W
		Present	Absent	2014	GO–588	Alexânia	16°07'13"S 48°29'08"W
		Absent		2003	DF–024	Ponte Alta	15°58'04"S 48°02'42"W
					DF–027	Ponte Alta	15°58'04"S 48°02'42"W
					DF–034	Valparaíso de Goiás	16°05'23"S 47°59'06"W
					DF–044	Valparaíso de Goiás	16°05'23"S 47°59'06"W
					DF–054	Ponte Alta	15°58'04"S 48°02'42"W
					DF–057	Ponte Alta	15°58'04"S 48°02'42"W
					GO–033	Luziânia	16°11'19"S 47°57'14"W
					GO–034	Luziânia	16°11'19"S 47°57'14"W
					GO–121	Goianápolis	16°30'24"S 49°01'02"W
					GO–126	Leopoldo de Bulhões	16°37'26"S 48°44'48"W
					GO–127	Leopoldo de Bulhões	16°37'26"S 48°44'48"W
					GO–204	Orizona	17°02'09"S 48°18'12"W
					GO–208	Orizona	17°02'09"S 48°18'12"W
					GO–211	Vianópolis	16°45'00"S 48°30'46"W
					GO–212	Vianópolis	16°45'00"S 48°30'46"W
					GO–218	Morrinhos	17°43'50"S 49°07'02"W

				2005	DF–154	Vargem Bonita	15°55'02"S 48°03'02"W
					DF–170	Núcleo Rural São José	15°42'30"S 47°21'56"W
					DF–209	Taquara	15°37'51"S 47°31'09"W
				2011	GO–495	Rajadinha	15°43'44"S 47°39'13"W
				2012	DF–487	Rajadinha	15°43'44"S 47°39'13"W
				2014	GO–589	Alexânia	16°04'51"S 48°30'24"W
				2015	GO–604	Bonfinopolis	16°35'26"S 48°59'52"W
					GO–605	Bonfinopolis	16°35'26"S 48°59'52"W
				2016	DF–663	Brazlândia	15°38'09"S 48°07'47"W
					GO–617	Anápolis	16°18'18"S 48°58'20"W
					GO–618	Anápolis	16°18'18"S 48°58'20"W
