# Supplementary material for: Geminiviridae and Alphasatellitidae Diversity Revealed by Metagenomic Analysis of Susceptible and Tolerant Tomato Cultivars across Distinct Brazilian Biomes
Source: Viruses. 2024 Jun 1;16(6):899. doi: 10.3390/v16060899 (PMC11209153; doi:10.3390/v16060899)
Supplement: Supplementary file 1 [file viruses-16-00899-s001.zip › Supplementary Table S2.pdf]

Table S2. List of species-specific primers used to detect different viruses and satellite DNA in tomato (Solanum lycopersicum) samples and details of information regarding the name of the primer, sequences, and annealing temperatures (AT ºC). Adapted from Reis et al. (2020).

Viral species / DNA segment	Primer name	Sequence 5'- 3'	AT (°C)
[1]*Tomato severe rugose virus* DNA–A	ToSRV-For5.1	AGCGTCGTTAGCTGTCTGGCA	58
	ToSRV-Rev5	TGCCGCAGAAGCCTTGAACGCACCT	
[1]*Tomato severe rugose virus* DNA–B	ToSRV-B-For	AAACCCACACGAAAGCAGAGTTT	55
	ToSRV-B-Rev	CACCACGTCTATACATATTGTCCAGG	
[1]*Euphorbia yellow mosaic virus* DNA–A	EuYMV-A-R-For	GGGGTTCCAAGTCCAATAAAGATGA	52
	EuYMV-A-R-Rev	CAGACACCTTATATTTGCCGGATTC	
[1]*Tomato chlorotic mottle virus* DNA–A	ToCMoV-A-For	TTTGGGCCGCTCTTTTGGG	47
	ToCMoV-A-Rev	CAAACTGAATGGGCCTTAAA	
[1]*Tomato chlorotic mottle virus* DNA–B	ToCMoV-B-For	GTATTTGTTCTGGGTGCAATCATAAAAC	55
	ToCMoV-B-Rev	TTGTACTAATGACACATTATTCAATCACGA	
[1]*Tomato golden vein virus* DNA–A	TGVV-A-For1	AAAGGAAGATAATTCAAATATAGGGA	51
	TGVV-A-Rev1	ATCTTCCTTTACTCACGTTCCTGAT	
[1]*Tomato golden vein virus* DNA–B	TGVV-B-S-For	CCCACTTTCCATAACCTACATGAGA	55
	TGVV-B-S-Rev	GGAGAGAAAATTGATAAGATCGGCATC	
[1]*Tomato mottle leaf curl virus* DNA–A	ToMoLCV-For	TGTGGTCCAGTCAATAAATG	47
	ToMoLCV-Rev	TGACTGGACCACATAGTAAA	
[1]*Sida micrantha mosaic virus* DNA–A	SiMMV-For	GATCTCGCTCCCCCCTCT	58
	SiMMV-Rev	AGATCGCACGACAACCAG	
[3]*Tomato yellow spot virus* DNA–A	F1A-ToY	ACGAAATCTTTTAGGAGCTAATGG	53
	R1A-ToY	CGTATTTCTGCAAAAAACTACTTCCT	
[3]*Tomato yellow spot virus* DNA–B	F3B-ToY	AATAAGGCGAAAGGTTAAAAGAATATGGCG	61
	R3B-ToY	GCCTTATTCACTTCACCTTCTTCGATTCAC	
[1]*Tomato yellow net virus* DNA–A	Abuti-A-For	GGACTCCAGGGGGCAAAA	55
	Abuti-A-Rev	AGTCCCGTCCGTACCACTTG	
[3]*Tomato chlorotic mottle Guyane virus* DNA–A	F3A-AM35	GGCGTATGAGTCGTTAGCTGATTGGC	62
	R4A-AM35	ATACGCCAAGGTCTTAAACTCAGAAACAA	
[3]*Tomato bright yellow mottle* virus DNA–A	F1A-TO167	CCCATTATTCCAAGGCCCAACAG	60
	R1A TO167	GGGCCTTTTTTATAGCAACTTAGC	
[3] *New species #1* DNA–A	F1A-C25	AAGTAAGGAAAAAATTCTTGGCTTGG	59
	R1A-C25	ATCCCAAGTGTTCCCTGACGAAAGAG	

[3] *New species #2* DNA–A	F6AC222	TATCAATTCGTCGTCTCCTGATTCCT	60
	R6AC222	AACTTTACCAAACTTAGTGACCAAG	
[3] *New species #3* DNA–A	F1A-C230	GTCTTTTGCTGTGTGGTCCAG	63
	R2A-C230	GCGGGTCGGGGGCATAAAAAAAT	
[3]New species #4 DNA–A	F1A-C16	TATGCTATGAATCGGTAGAACGG	58
	R1A-C16	GCATAAGTTTTCTCTGAATCCC	
[3]*New species #5* DNA–A	F2A-C12	GATTGTGTCCTGGGCGGTTATTTATTTCG	63
	R2A-C12	GTACAACACAGAGCTGCTAAAAAAACGAGG	
[1]Alfasatellites	Alfa-For	TGGTGTCCTGGCTTATAT	46
	Alfa-Rev	GGCGGAGTCCTTTTTTTT	
[2]*Tomato associate geminivirus1*	Cap2KpnI-F	GGTACCCCCCTTGGAAATGTAGTCTGCAAC	66
	Cap2KpnI-R	GGTACCTTTGAGGAGAGAGGTATACTTCG	
[2]*Tomato apical leaf curl virus*	Cap1PstI-F	CTGCAGAYTTGCGCGGATCGATTAAT	68
	Cap1PstI-R	CTGCAGAAATGCGTTGTAACTTCTCGGATAT	

[1]Primers designed by Reis et al. [2020]; [2]Primers designed by Batista et al. [2019]; [3]Primers designed in the present study.
