# Supplementary figures and images for: Geminiviridae and Alphasatellitidae Diversity Revealed by Metagenomic Analysis of Susceptible and Tolerant Tomato Cultivars across Distinct Brazilian Biomes
Source: Viruses. 2024 Jun 1;16(6):899. doi: 10.3390/v16060899 (PMC11209153; doi:10.3390/v16060899)

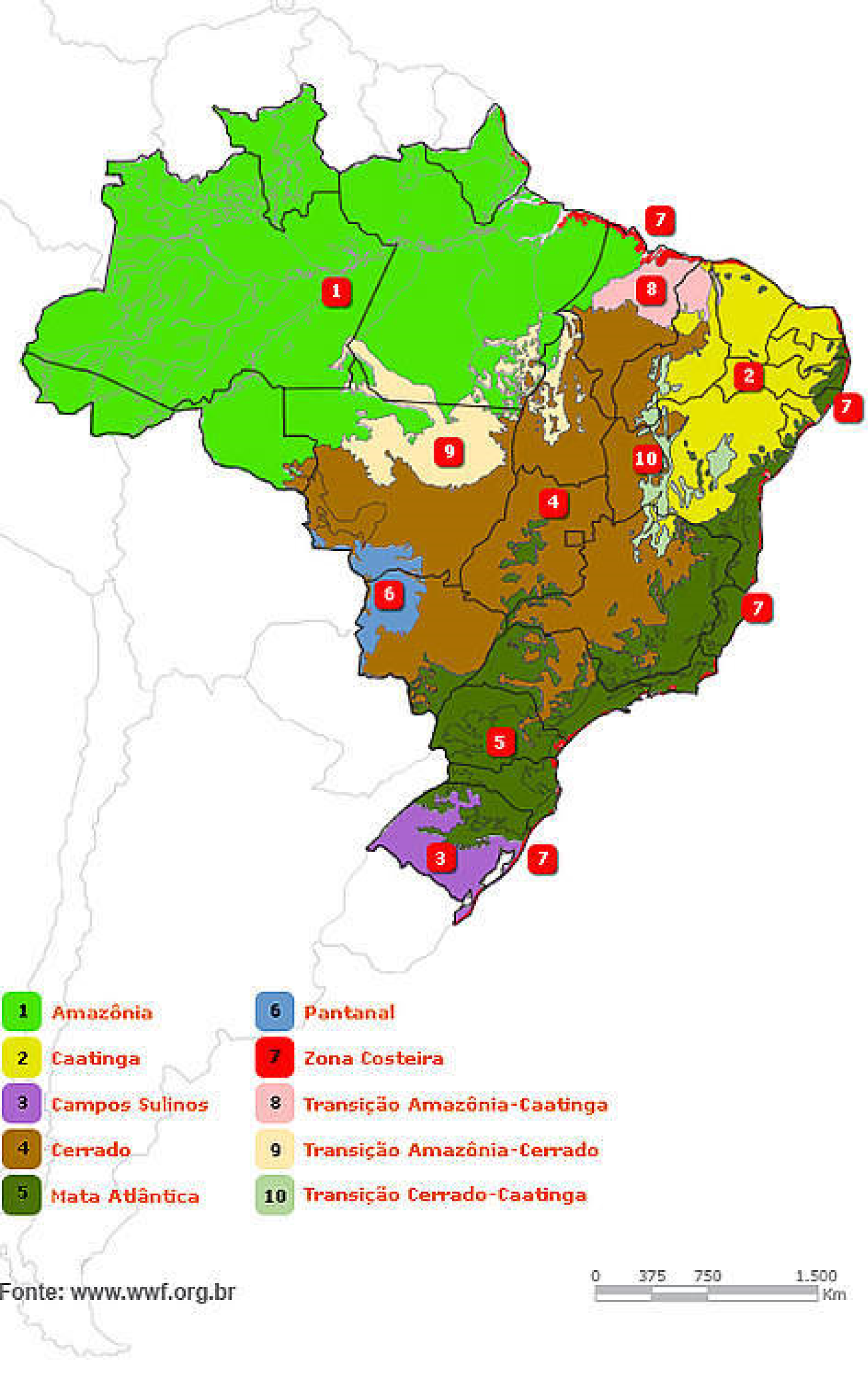

Supplement: Supplementary file 1 [file viruses-16-00899-s001.zip › Supplementary Figure S1.tif]
